# Lea’s Series of Medical Epitomes—Manton’s Orstetrics

**Published:** 1903-10

**Authors:** 


LEA'S SERIES OF MEDICAL EPITOMES—MANTON'S OBSTETRICS.—A Manual of Obstetrics for Students and Practitioners. By W. P. Manton, M. D., Adjunct-Professor of Obstetrics and Professor of Clinical Gynecology, Detroit College of Medicine. In one 12mo volume of 265 pages, with 82 illustrations. Cloth, $1.00 net. Lea Brothers & Co., Publishers, Philadelphia and New York. 1903.

A surprising amount of important information is contained in the 265 pages of this little manual. The book will prove to be a boon to the overworked medical student and to the graduate who finds it necessary to refresh his memory on the essentials of Obstetrics in making preparation for State board and other examinations. R. & L.

